# Intratumoral Heterogeneity, Its Contribution to Therapy Resistance and Methodological Caveats to Assessment

**DOI:** 10.3389/fonc.2014.00142

**Published:** 2014-06-10

**Authors:** Mirjam Renovanz, Ella L. Kim

**Affiliations:** ^1^The Translational Neurooncology Research Group, Department of Neurosurgery, Johannes Gutenberg University Medical Centre, Mainz, Germany

**Keywords:** cancer, intratumor heterogeneity, anti-cancer therapy, tumor recurrence

Cancer is one of the most urgent health issues of today. According to WHO, the number of cancer cases is expected to increase by 75% in the next two decades (1). Despite some remarkable achievements in the fields of cancer prevention and early detection, the goal of developing effective anti-cancer therapies still remains unmet. Tumor recurrence due to treatment resistance is the most common cause of death from cancer. Delineating cellular and molecular mechanisms underlying tumor recurrence is of prime importance for the ability to improve the efficacy of existing therapies and develop new strategies to cancer treatment.

The aim of any anti-cancer treatment is to selectively kill cancer cells by targeting key biological properties essential for the maintenance of tumorigenicity and malignant progression (2). Currently, cytotoxic therapies are still a mainstay of cancer treatment that relies heavily on radiation treatment and chemotherapy. Even though cytotoxic treatments can be effective in some types of cancers, the clinical experience accumulated over the past few decades indicates that conventional cytotoxic therapy may not suffice to achieve a satisfactory level of the therapeutic efficacy. A conceptual framework for cytotoxic therapies derives from the observation that there is a direct relationship between proliferation rate and cytotoxic sensitivity, implying that rapidly dividing cancer cells rather than largely quiescent normal cells should be preferentially targeted by cytotoxic agents. However, proliferation rates of tumor cells can vary in a broad range between and within tumors. This is thought to be one of the reasons for insufficient efficacy of cytotoxic therapies (3). Furthermore, cancer cells can neutralize the effects of cytotoxic treatments by utilizing a plethora of often overlapping mechanisms that include aberrant DNA repair and cell death pathways, drug efflux, hypoxia-induced apoptosis resistance and invasion, alterations in drug metabolism, unfolded protein response, and autophagy [reviewed in Ref. (4, 5)].

A newer type of anti-cancer therapy generally called molecularly targeted therapy relies on rationally designed agents to target, with a high degree of specificity, well-defined molecules or pathways that operate in cancer cells to maintain their malignant potential. Although both cytotoxic and molecularly targeted therapeutic approaches generally exploit differences between neoplastic and normal cells, only targeted therapies enable the so-called precision medicine. Recent advances in the field of molecular profiling have opened up a real possibility to make better informed treatment decisions based on the data from personalized tumor profiling [reviewed in Ref. (6, 7)]. However, despite some remarkable successes of targeted therapies (8, 9), their utility in advanced cancers has so far been limited due to an almost inevitable tumor recurrence even after successful initial response [reviewed in Ref. (6, 10, 11)]. The escape mechanisms underlying the inherent and acquired resistance to targeted therapies include feedback activation of signaling pathways with redundant functions (12), co-occurrence of mutations in other genes involved in synergistic interactions with the target gene (13), or emergence of subclones with secondary mutations coding for resistant versions of drug targets (14). Global profiling of cancer genomes has enabled the stratification of major cellular pathways involved in the development of therapeutic resistance in different types of cancer. Providing a molecular explanation of the limited efficacy of targeted monotherapies, cancer genomics studies reveal a high degree of functional redundancy between oncogenic driving events [reviewed in Ref. (7, 15)].

New generation sequencing methodologies while enabling to identify genomic alterations associated with different types of cancer with an unprecedented completeness also revealed the high degree of genetic diversity existing not only between different types of cancer but also between individual tumors of the same histotype [reviewed in Ref. (16, 17)]. A broad range of phenomena encompassed in the term “tumor heterogeneity” include (epi) genetic, phenotypic, and gene expression pattern diversity across different types of cancer, between different tumors of the same histotype (interpatient heterogeneity), between different tumors from the same patient (primary tumor or metastasis), or within the same tumor (intratumor heterogeneity). Intratumor heterogeneity manifests in spatial and temporal patterns of genetic, phenotypic, and functional diversity (18). There is a growing evidence of intratumor heterogeneity in different types of cancers including breast cancer (19), renal carcinomas (20, 21), and glioblastomas (22). Mechanisms underlying intratumor heterogeneity can be broadly divided into those that are powered by genomic instability or non-mutational mechanisms. The latter include stochastic variations in cellular responses between genetically identical tumor cells, modulation of cellular responses by tumor microenvironment, and/or phenotypic and functional plasticity contributed by cancer stem cells (CSCs) [reviewed in Ref. (17, 23)]. Genomic instability defined as progressive mutagenic process accompanying neoplastic growth is the major mechanism of generating new mutations. Less well-characterized mechanisms include genome doubling (24) and rare cataclysmic genomic rearrangements resulting in massive genomic rearrangements (25). According to the clonal evolution model, persistent changes in tumor genomes generate genetically and functionally distinct clones that may occupy different geographic territories within the tumor. There are many lines of evidence for the spatial patterns of intratumor heterogeneity in advanced cancers. In glioblastomas (glioblastoma multiforme, GBM), distinct patterns of genomic alterations and gene expression signatures can be found in different regions within the same tumor (22). Strikingly, molecular signatures that were previously thought to be associated with clinically distinct subtypes of GBM (26, 27) were found to co-exist within the same tumor (22). Similarly, more than 60% of all somatic mutations identified through a multi-region genetic analysis in renal carcinoma were found spatially separated within the same tumor and not detectable in every tumor region analyzed (20). These findings indicate that different sampling strategies can strongly impact the interpretation of molecular profiling data obtained with single tumor samples and emphasize the need for suitable methodologies that would take into account the spatiotemporal patterns of intratumor heterogeneity.

These considerations are of particular relevance in the context of the CSC hypothesis, which postulates that CSCs constitute only a minor fraction of tumor cells capable of initiating tumor growth [reviewed in Ref. (1, 28)]. In light of the findings that different types of tumor cells can be geographically separated within the tumor (20, 22), it is possible that CSCs may be unevenly distributed throughout the tumor. It should be noted that in many studies, the tumorigenic potential is compared between CSCs and non-CSC tumor cells isolated from a single tumor region. Thus, the relative proportion of CSCs may vary not only between different tumor types (CSC-derived malignancies vs. non-CSC tumors) but also within the tumors that comply with the CSC paradigm, depending on the tumor region analyzed. The CSC hypothesis postulates that CSC is the only type of tumor cells (in CSC-derived tumors) that possesses the propensity to initiate and maintain tumor growth (29, 30). However, in the light of consideration that a single tumor region may not be representative of the whole tumor (20, 22), it cannot be excluded that highly tumorigenic non-CSC may have been missed in analyses using single tumor specimens. In such a case, the conclusion that non-CSCs have generally lower tumorigenicity compared to CSCs would have been misleading due to a sampling bias.

The fact that genetically (and functionally) heterogeneous types of cancer cells can be separated spatially within a tumor raises several important questions concerning the identity of tumor clones that are capable of escaping from anti-cancer treatments and repopulating the tumor. There is some evidence that exposure to therapy may influence the dynamics of clonal repopulation and lead to the alternation of clonal dominance as a consequence of treatment. For example, by applying next generation sequencing to compare somatic mutations in matched pairs of *de novo* and recurrent AMLs, it was established that a minor AML clone underrepresented in the primary tumor became dominant in recurrent tumors as a consequence of chemotherapy (31). Similarly, cytogenetics and gene expression analyses in a series of sequential samples of multiple myeloma from the same patient treated with different chemotherapy regimens have revealed that tumor relapse was associated with the preferential outgrowth of a minor clone (32). In the emerging scenario, the dominance of clone A in untreated tumors can be lost during anti-cancer therapy (provided that clone A fulfils the criteria for the target cell) whereas clone B lacking the molecular target can become dominant even if it was underrepresented in untreated tumors.

The realization that intratumor heterogeneity poses one of the major challenges to overcome resistance to anti-cancer therapy raises a number of questions: are there common molecular denominators underlying resistance to different types of therapy? Is there an interaction between different populations of cancer cells residing in the same or different geographic regions of the same tumor? What is the impact of different types of anti-cancer therapy in the emergence of resistant clones? To address these issues, there is a need of suitable methodologies that would take into account the spatiotemporal patterns of intratumoral diversity. It has been proposed that multiple sampling analyses of multiple regions from matched pairs of untreated and recurrent tumors would be required to assess the impacts of intratumoral diversity on the development of resistance to anti-cancer therapies (22). Such an approach may have limited applicability in those tumors for which serial sampling is difficult to achieve. For example, serial tumor sampling in post-operated GBM is likely to be a challenge considering that repeat surgery, as a treatment option, is possible only for 15–45% of patients depending on age, neurologic performance, and extent of resection during the first operation (33). Considering that multisampling is a much more realistic task during the first surgery, a combined approach based on establishing heterogeneous primary cultures from multisampled untreated tumors and selecting from them therapy-resistant clones *in vitro* might be more feasible. Such an approach has the advantage of reducing the variability in treatment conditions and dissecting the effects of single and combined treatments. By comparing treatment responses in different types of cancer cells from the same tumor should allow to improve predictions on the efficacy of a particular treatment scheme in a particular tumor.

It should be noted that the degree of intratumoral heterogeneity may not necessarily reflect an enhanced malignant potential. It is believed that a considerable portion of new mutations arising in the course of tumor evolution are passenger mutations (7). In such a case, the number of clinically relevant oncogenic driver mutations may still be within the range attackable by combinatorial treatment regimens using different therapies applied either simultaneously or sequentially. Also, the growing realization that tumor growth before, during, or after treatment can be driven by molecularly distinct populations of cells (31, 32) may have important implications for the rational design of combinatorial therapy regimens that would match the dynamically changing cellular and molecular composition of the tumor. Unfortunately, increased toxicity poses a general problem impeding the benefits of combined therapies. In this regard, alternating targeted therapies using agents with non-overlapping toxicity profiles may provide a means to achieve additive anti-tumor effects without increasing overall toxicity. The efficacy of alternating therapies guided by “real-time” molecular assessments has been demonstrated for metastatic lung tumor originating from adenocarcinoma of the tongue (34). In this study, a clinical benefit could be reached by applying alternating treatments with different therapeutic agents whose effectiveness was inferred by comparing whole-genome and RNA profiles of untreated and recurrent tumors.

The emerging scenario of recurrent tumor growth reveals key roles of intratumoral heterogeneity in intrinsic and acquired resistance to cytotoxic and targeted therapies. Understanding spatiotemporal patterns and dynamics of intratumoral heterogeneity before and during therapy is crucial for the ability to design individual-tailored treatment regimens best suited to a particular molecular context.

## Conflict of Interest Statement

The authors declare that the research was conducted in the absence of any commercial or financial relationships that could be construed as a potential conflict of interest.

## References

[1] Adams JM Kelly PN Dakic A Carotta S Nutt SL Strasser A Role of “cancer stem cells” and cell survival in tumor development and maintenance. Cold Spring Harb Symp Quant Biol (2008) 73:451–9 10.1101/sqb.2008.73.004 19022754

[2] Hanahan D Weinberg RA Hallmarks of cancer: the next generation. Cell (2011) 144:646–74 10.1016/j.cell.2011.02.013 21376230

[3] Mitchison TJ The proliferation rate paradox in antimitotic chemotherapy. Mol Biol Cell (2012) 23:1–6 10.1091/mbc.E10-04-0335 22210845 PMC3248889

[4] Masui K Gini B Wykosky J Zanca C Mischel PS Furnari FB A tale of two approaches: complementary mechanisms of cytotoxic and targeted therapy resistance may inform next-generation cancer treatments. Carcinogenesis (2013) 34:725–38 10.1093/carcin/bgt086 23455378 PMC3616676

[5] Kastan MB DNA damage responses: mechanisms and roles in human disease: 2007 G.H.A. Clowes memorial award lecture. Mol Cancer Res (2008) 6:517–24 10.1158/1541-7786.MCR-08-0020 18403632

[6] Bernards R A missing link in genotype-directed cancer therapy. Cell (2012) 151:465–8 10.1016/j.cell.2012.10.014 23101617

[7] Vogelstein B Papadopoulos N Velculescu VE Zhou S Diaz LA Jr Kinzler KW Cancer genome landscapes. Science (2013) 339:1546–58 10.1126/science.1235122 23539594 PMC3749880

[8] Chapman PB Hauschild A Robert C Haanen JB Ascierto P Larkin J Improved survival with vemurafenib in melanoma with BRAF V600E mutation. N Engl J Med (2011) 364:2507–16 10.1056/NEJMoa1103782 21639808 PMC3549296

[9] O’Brien SG Guilhot F Larson RA Gathmann I Baccarani M Cervantes F Imatinib compared with interferon and low-dose cytarabine for newly diagnosed chronic-phase chronic myeloid leukemia. N Engl J Med (2003) 348:994–1004 10.1056/NEJMoa022457 12637609

[10] Blair BG Bardelli A Park BH Somatic alterations as the basis for resistance to targeted therapies. J Pathol (2014) 232:244–54 10.1002/path.4278 24114654

[11] Logue JS Morrison DK Complexity in the signaling network: insights from the use of targeted inhibitors in cancer therapy. Genes Dev (2012) 26:641–50 10.1101/gad.186965.112 22474259 PMC3323875

[12] Prahallad A Sun C Huang S Di Nicolantonio F Salazar R Zecchin D Unresponsiveness of colon cancer to BRAF(V600E) inhibition through feedback activation of EGFR. Nature (2012) 483:100–3 10.1038/nature10868 22281684

[13] Mao M Tian F Mariadason JM Tsao CC Lemos R Jr Dayyani F Resistance to BRAF inhibition in BRAF-mutant colon cancer can be overcome with PI3K inhibition or demethylating agents. Clin Cancer Res (2013) 19:657–67 10.1158/1078-0432.CCR-11-1446 23251002 PMC3563727

[14] Sakai W Swisher EM Karlan BY Agarwal MK Higgins J Friedman C Secondary mutations as a mechanism of cisplatin resistance in BRCA2-mutated cancers. Nature (2008) 451:1116–20 10.1038/nature06633 18264087 PMC2577037

[15] Stratton MR Campbell PJ Futreal PA The cancer genome. Nature (2009) 458:719–24 10.1038/nature07943 19360079 PMC2821689

[16] Mardis ER Genome sequencing and cancer. Curr Opin Genet Dev (2012) 22:245–50 10.1016/j.gde.2012.03.005 22534183 PMC3890425

[17] Meacham CE Morrison SJ Tumour heterogeneity and cancer cell plasticity. Nature (2013) 501:328–37 10.1038/nature12624 24048065 PMC4521623

[18] Yap TA Gerlinger M Futreal PA Pusztai L Swanton C Intratumor heterogeneity: seeing the wood for the trees. Sci Transl Med (2012) 4:127s110 10.1126/scitranslmed.3003854 22461637

[19] Russnes HG Navin N Hicks J Borresen-Dale AL Insight into the heterogeneity of breast cancer through next-generation sequencing. J Clin Invest (2011) 121:3810–8 10.1172/JCI57088 21965338 PMC3195464

[20] Gerlinger M Rowan AJ Horswell S Larkin J Endesfelder D Gronroos E Intratumor heterogeneity and branched evolution revealed by multiregion sequencing. N Engl J Med (2012) 366:883–92 10.1056/NEJMoa1113205 22397650 PMC4878653

[21] Martinez P Birkbak NJ Gerlinger M McGranahan N Burrell RA Rowan AJ Parallel evolution of tumour subclones mimics diversity between tumours. J Pathol (2013) 230:356–64 10.1002/path.4214 23716380

[22] Sottoriva A Spiteri I Piccirillo SG Touloumis A Collins VP Marioni JC Intratumor heterogeneity in human glioblastoma reflects cancer evolutionary dynamics. Proc Natl Acad Sci U S A (2013) 110:4009–14 10.1073/pnas.1219747110 23412337 PMC3593922

[23] Burrell RA McGranahan N Bartek J Swanton C The causes and consequences of genetic heterogeneity in cancer evolution. Nature (2013) 501:338–45 10.1038/nature12625 24048066

[24] Carter SL Cibulskis K Helman E McKenna A Shen H Zack T Absolute quantification of somatic DNA alterations in human cancer. Nat Biotechnol (2012) 30:413–21 10.1038/nbt.2203 22544022 PMC4383288

[25] Stephens PJ Greenman CD Fu B Yang F Bignell GR Mudie LJ Massive genomic rearrangement acquired in a single catastrophic event during cancer development. Cell (2011) 144:27–40 10.1016/j.cell.2010.11.055 21215367 PMC3065307

[26] Verhaak RG Hoadley KA Purdom E Wang V Qi Y Wilkerson MD Integrated genomic analysis identifies clinically relevant subtypes of glioblastoma characterized by abnormalities in PDGFRA, IDH1, EGFR, and NF1. Cancer Cell (2010) 17:98–110 10.1016/j.ccr.2009.12.020 20129251 PMC2818769

[27] Chen R Nishimura MC Bumbaca SM Kharbanda S Forrest WF Kasman IM A hierarchy of self-renewing tumor-initiating cell types in glioblastoma. Cancer Cell (2010) 17:362–75 10.1016/j.ccr.2009.12.049 20385361

[28] Visvader JE Lindeman GJ Cancer stem cells in solid tumours: accumulating evidence and unresolved questions. Nat Rev Cancer (2008) 8:755–68 10.1038/nrc2499 18784658

[29] Garvalov BK Acker T Cancer stem cells: a new framework for the design of tumor therapies. J Mol Med (Berl) (2011) 89:95–107 10.1007/s00109-010-0685-3 20890588

[30] Magee JA Piskounova E Morrison SJ Cancer stem cells: impact, heterogeneity, and uncertainty. Cancer Cell (2012) 21:283–96 10.1016/j.ccr.2012.03.003 22439924 PMC4504432

[31] Ding L Ley TJ Larson DE Miller CA Koboldt DC Welch JS Clonal evolution in relapsed acute myeloid leukaemia revealed by whole-genome sequencing. Nature (2012) 481:506–10 10.1038/nature10738 22237025 PMC3267864

[32] Keats JJ Chesi M Egan JB Garbitt VM Palmer SE Braggio E Clonal competition with alternating dominance in multiple myeloma. Blood (2012) 120:1067–76 10.1182/blood-2012-01-405985 22498740 PMC3412330

[33] Sia Y Field K Rosenthal M Drummond K Socio-demographic factors and their impact on the number of resections for patients with recurrent glioblastoma. J Clin Neurosci (2013) 20:1362–5 10.1016/j.jocn.2013.02.010 23769599

[34] Jones SJ Laskin J Li YY Griffith OL An J Bilenky M Evolution of an adenocarcinoma in response to selection by targeted kinase inhibitors. Genome Biol (2010) 11:R82 10.1186/gb-2010-11-8-r82 20696054 PMC2945784

